# HEDGES co-prevents both SARS-CoV-2 and pandemic influenza infection in mice by rapid, durable co-production of twelve different anti-pandemic monoclonal antibodies

**DOI:** 10.1371/journal.pone.0309923

**Published:** 2026-01-23

**Authors:** Marissa Mack, Alice Ye, Sarah Ursu, Ryan Ice, Liliana Soroceanu, Stan Shoor, Sean McAllister, Tim Heath, Chakkrapong Handumrongkul, Robert Debs

**Affiliations:** DNARx, San Francisco, California, United States of America; Waseda University: Waseda Daigaku, JAPAN

## Abstract

Despite all currently available anti-pandemic monoclonal-antibodies (mAbs) and vaccines, subsequently emerging pandemic-infections will likely become more pan-resistant-, -transmissible and/or -lethal. We have created HEDGES generation-2, a significantly more-combinatorial, -synergistic version of our generation-1 HEDGES DNA vector-based platform. We previously published that one safe intravenous injection of a HEDGES generation-1 DNA vector encoding one of three different FDA-approved mAbs produced durable therapeutic serum mAb levels as well as critical therapeutic endpoints in immunocompetent mice. Here we show one safe, intravenous administration of a 2^nd^-generation HEDGES DNA vector co-encoding four different anti-SARS-CoV-2 mAbs rapidly then durably co-produces high anti-SARS-CoV-2 mAb serum levels that effectively block SARS-CoV-2 virus binding to the ACE-2 spike protein in immunocompetent mice. In addition, four weekly intravenous HEDGES generation-2 DNA vector administrations co-encoding a total of ten-different anti-SARS-CoV-2 mAbs, 5J8, plus an anti-1918 pandemic influenza mAb and mepolizumab, an FDA-approved anti-IL-5 mAb, durably co-produce highly-neutralizing 5J8 anti-pandemic influenza mAb serum levels, as well as durably block SARS-CoV-2 virus-ACE-2 receptor binding in mice. Furthermore, unlike vaccines and mAbs, HEDGES does not require an intact cold chain and is readily freeze dried, enabling its prolonged storage at ambient temperatures worldwide, even in equatorial regions. Also, HEDGES can create, then deploy novel, more effective anti-pandemic mAbs ~three weeks after their identification. Conversely, vaccines require ~three months to deploy, recombinant-mAbs ~nine months. By rapidly then durably co-producing many different highly-neutralizing, highly-synergistic anti-pandemic mAbs, HEDGES may effectively co-prevent both SARS-CoV-2 and pandemic-influenza infections. HEDGES may also prevent even more-transmissible, -pan-resistant and/or -lethal pandemic diseases that subsequently-emerge.

## Introduction

Current as well as future pandemics appear likely to become more-frequent, more-transmissible, and/or more-lethal [1,2]. For-example, emergence of progressively more pan-resistant SARS-CoV-2 Omicron escape mutant-strains has now rendered some SARS-CoV-2 escape mutant strains pan-resistant to all-available anti-pandemic r-mAbs as well as to all vaccines [3] Specifically,1^st^-generation anti-SARS-CoV-2 r-mAbs and vaccines effectively prevented symptomatic SARS-CoV-2 Delta-strain infection [4,5]. Within 2-years, no anti-SARS-CoV-2 mAbs or vaccines could prevent symptomatic infection with now-pan-resistant Omicron escape-mutant strains [6]. Although Omicron remains less-lethal than Delta, more-lethal Omicron escape-mutant strains exhibiting MERS-like ~35% lethality may subsequently-emerge [7]. In-addition, novel, even more-lethal pandemic diseases may subsequently-emerge at any time [1,2]. Again, mortality may exceed 35% [7], thus devastating the world. Previously, 50-100 million-people died within months of the 1918-pandemic influenza-pandemic emerging [8]. Mean-age of those dying from SARS-CoV-2 is ~85 years-old, mean-age of those dying from 1918-pandemic-influenza was ~28 years-old [8].

Several clinical observations have identified critical limitations of all now available anti-SARS-CoV-2 r-mAbs. Specifically, previously FDA-approved combinations of 2-different anti-SARS-CoV-2 r-mAbs that effectively prevented symptomatic SARS-CoV-2 Delta-strain infections [4,5] are now ineffective in preventing symptomatic pan-resistant Omicron escape mutant strain infection [6]. In addition, r-mAbs must be re-administered every 3-weeks, thus imposing additional costs and logistical constraints [4]. Furthermore, whereas new HEDGES-based anti-pandemic mAbs require only ~three weeks to create then deploy, new-vaccines require ~3-months to create-then-deploy [9], new r-mAbs require ~9-months to create then deploy [10].

DNARx previously reported the creation of its HEDGES (High-level Extended Duration Gene Expression System) generation-1 intravenous (IV), nonviral, DNA-vector based gene therapy platform [11]. HEDGES neither detectably integrates into genomic DNA, induces adaptive immune responses, nor elicits anti-vector targeted immune responses that prevent effective re-dosing in immunocompetent hosts [11]. In addition, critical rodent toxicity markers remain near or at background levels [11]. Specifically, one IV HEDGES DNA-vector administration into immunocompetent mice safely, rapidly, and durably produces therapeutic serum levels of one or more cDNA encoded human proteins. These proteins include durably producing the FDA-approved human cytokine, hG-CSF (protein T^1^/_2_ ~ 2 hours [11,12]). HEDGES also produces durable therapeutic serum levels of 5J8, an anti-1918 pandemic influenza mAb [13], Rituximab, an anti-human CD20 mAb [14], and Mepolizumab, an anti-human IL-5 mAb [15]. HEDGES DNA vector-encoded genes are expressed in ~35% of all lung vascular endothelial cells [11]. In the absence of inflammation, normal vascular endothelial cells remain largely nondividing. As HEDGES DNA vectors do not detectably integrate into genomic DNA [11], they remain episomal. This combination of features accounts for HEDGES ability to durably produce its cDNA encoded proteins [11].

Here, we sought to create a significantly more combinatorial, synergistic 2^nd^-generation of our original HEDGES generation-1 DNA-vector based platform [11]. A new platform that effectively, rapidly, and durably co-produces large combinations of different, highly synergistic, highly neutralizing anti-pandemic mAbs. To accomplish this, we chose the cPASS assay [16], which quantitates inhibition of SARS-CoV-2-RBD binding to the host cell ANC2 receptor in *ex vivo* mouse serum. We chose this ELISA-based SARS-CoV-2-ACE2 receptor binding assay because cPASS results strongly correlate with results obtained using each of several different direct *in vivo* SARS-CoV-2 virus neutralization assays [16–19].

Our HEDGES-generation-2 approach may also effectively co-prevent pandemic influenza [13], HIV [20–22], and malaria [23] infection, even in severely immunosuppressed individuals. This 2^nd^-generation HEDGES DNA-vector based approach may also effectively co-prevent even more transmissible, pan-resistant and/or lethal pandemic infections that may subsequently emerge at any time [1,2].

## Materials and methods

### Mice

All mice used were female outbred Hsd:ICR (CD-1 ®) mice from Envigo. All studies were conducted in accordance with protocols approved by the Institutional Animal Care and Use Committee at the California Pacific Medical Center Research Institute. A control group of un-injected, ~ 25 gm CD-1 female mice obtained from Envigo were included with all the test groups in every experiment presented in this manuscript. In every experiment performed, values obtained from control mice did not statistically significantly differ from assay background levels.

### Plasmid construction

The plasmids were constructed as previously described^16^. All cDNA for the anti-SARS-CoV-2 mAbs [24–28], ACE-2, GH, and GLA were ordered from GeneArt (Thermo Scientific) as codon optimized CpG-free gene fragments and inserted into a HEDGES Expression plasmid vector at the BstEII and BglII sites. Dual expression cassettes were generated by excising the first cassette out of a puc-19 based cloning vector at EcoRI and XbaI sites and inserting it into the second vector at the EcoRI and NheI sites. This method was repeated to generate the three and four expression cassette plasmids. Plasmids containing 5J8, aIL5, aCD20, and G-CSF were constructed as previously described [11].

### Plasmid production

All plasmids were produced using the Qiagen EndoFree Maxi kit (Qiagen: 12362) according to the manufacturer’s protocol. The final plasmid was dissolved in lactated ringer’s solution (LRS).

### Liposomes

DOTAP (18:0 TAP, 1,2-dioleoyl-3-trimethylammonium-propane (chloride salt)) and DMPC (14:0 PC 1,2-dimyristoyl-sn-glycero-3-phosphocholine) lipids were acquired from Avanti Polar Lipids, (SKU 890890C and 859345, respectively).

### HEDGES injections

**Fig 1:**
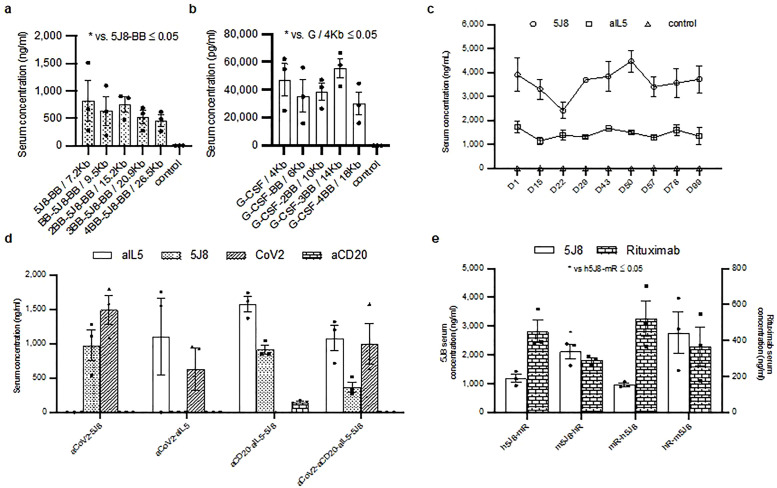
Optimization of the HEDGES vector size and orientation. **a-b** ELISA values from 5J8 (**a**) and G-CSF (**b**) specific ELISAs. Mice were injected with 75 µg of the indicated plasmid in groups of 3 and serum was collected 24 hours an analyzed via specific ELISA. Data is presented as mean ± SEM. BB refers a non-coding insulator sequence. **c** ELISA values from 5J8 and aIL5 specific ELISAs from 3 mice injected with a single DNA vector encoding both aIL5 and 5J8. Serum was collected 24 hours later and then every 7 to 14 days until day 99. Data is presented as mean ± SEM. **d** Antibody concentration level from mouse serum detected by specific ELISAs for aIL5 (Mepolizumab), 5J8, aCD20 (Rituximab), and aCoV-2. Mice were injected in groups of 3 with the plasmid as labeled on the x-axis. Expression was measured 24 hours after injection. Data is presented as mean ± SEM. **e** ELISA values from 5J8 and Rituximab specific ELISAs measuring the protein expression levels of mice in groups of 3 following injection with the indicated plasmids. The plasmids contain either human (h) or murine (m) enhancers in the 3’ or 5’ orientation. For all panels data is presented as mean ± SEM, n = 3. Statistical significance (p ≤ 0.05) as calculated by 2-tail t-test is indicated by asterisk, no asterisk indicates a non-significant difference.

**Fig 2:**
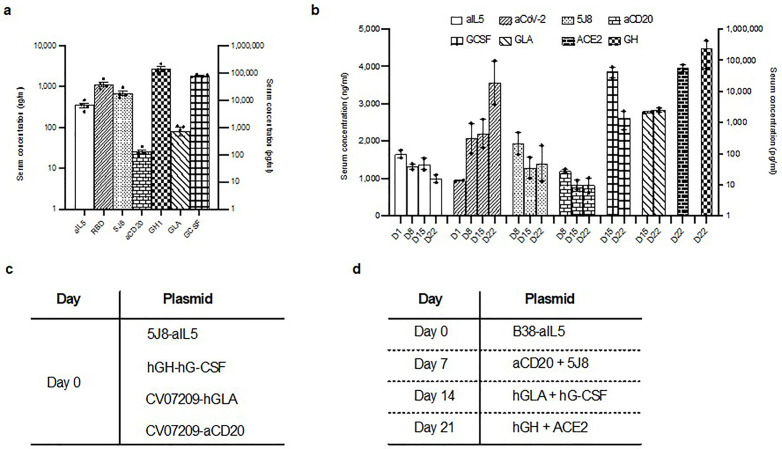
Co-injection versus repeat injection of HEDGES DNA vectors to maximize durable co-production of up to seven different transgene cDNA encoded human proteins. **a** ELISA values from specific ELISAs as measured after IV HEDGES injection Mice were injected in groups of 3 and data is presented as mean ± SEM. **b** ELISA values for the mAbs (aIL5, aCoV-2, 5J8, and aCD20) are represented on the left y-axis in ng/ml and the ELISA values for the non-mAb proteins (G-CSF, GH, ACE-2, and GH) are represented on the right y-axis in pg/ml. Mice were injected in groups of 3 and data is presented as mean ± SEM **c** ELISA values as measured after IV HEDGES injection. The values for aCoV-2, aIL5, and 5J8 are represented on the left y-axis in ng/ml while values for GH, G-CSF, and GLA are represented on the right y-axis in pg/ml. The x-axis represents different DNA dose-lipid ratios as indicated. DNA dose in µg is listed first and lipid dose in nmol is second. Experiment 102/1400 is 102 µg plasmid and 1400 nmol lipid. Mice were injected in groups of 4 and data is presented as mean ± SEM. Statistical significance (p ≤ 0.05) as calculated by 2-tail t-test is indicated by asterisk, no asterisk indicates a non-significant difference. **c, d** Injection schedules for panels a (c) and b (d). The plasmids as indicated on the right correspond to the day they were injected on the left hand side. Panel a was injected with one injection, whereas panel b were injected over the course of 4 weeks.

Mice were injected in the lateral tail vein with liposomes suspended in lactated ringer’s solution (LRS). Two minutes later, the mice were injected in the lateral tail vein with the DNA vectors in LRS. All mice were injected with 1000 nmol DOTAP combined with 1000 nmol DPMC, except for the following: Fig 1a, 1b and 1e (1120 nmol) and Fig 2a (1300 nmol). The DNA and lipid doses for Fig 2c are indicated by the x-axis label (DNA µg/lipid nmol). The DNA dose for the remaining experiments is 90 µg per mouse except Fig 2a (75 µg).

### Serum collection

Mice were anesthetized with isoflurane inhalation and bled via submandibular vein puncture. The blood was collected into serum separator tubes (365967, Becton Dickinson) [11].

### ELISA

For the hIgG specific ELISA, immunoassay plates were coated with goat anti-human IgG Fc capture antibody (Bethyl laboratories: A80-104A) at 2 µg/ml overnight and blocked with 2% bovine serum albumin. The standard protein used was normal human IgG (BioRad: 5172−9017). The samples were detected with goat anti-human IgG Fc, HRP (Millipore: AP113P) at 1:20000. The plates were developed using TMB ultra substrate (Fisher: PI34029). The absorbance at 450nm was measured on a BMG Labtech spectrostar nano plate reader and the standard curve was analyzed by 4PL using the MARS data analysis software. The aCoV-2 specific ELISA was run in the same manner with SARS-CoV-2 Spike Protein, RBD (Genscript: Z03479) as the coating protein and goat anti-human IgG Fc, HRP (Millipore: AP113P) detection antibody. The standard protein used was the mAb CV07209 expressed in ExpiCHO (Thermo Fisher: A29133) purified with Protein G columns (Thermo Fisher: 89927) as previously described [11]. The ELISAs for G-CSF, 5J8, aIL5, and Rituximab were run as previously described [11]. GLA ELISA was from RayBiotech (ELH-aGLA) and run as directed. The GH ELISA used anti-GH1 capture antibody (Abcam: ab64499) with recombinant GH1 (Abcam: ab116162) as standard and anti-GH1 HRP detection antibody (Abcam: 106749). All serum was diluted at 1:100.

### cPass assay

All surrogate neutralization data was generated via SARS-CoV-2 surrogate neutralization antibody detection kit, cPass (Genscript, NJ) following the provided protocol. Briefly, diluted serum was mixed with diluted RBD-HRP and incubated at 37°C for 20 minutes before 100 µl was added to the provided ACE-2 coated 96-well plate. The plate was then incubated at 37°C for 20 minutes before washing and development with the provided TMB. The absorbance at 450nm was measured on a BMG Labtech spectrostar nano plate reader. The surrogate neutralization was calculated as 1 minus the ratio of sample OD to negative control OD, presented as a percentage. Serum was diluted 1:10 for experimental samples, while the serum for the IC50 calculation and dose-response curve were serially diluted 1:10, 1:25, 1:50, 1:75, and 1:100.

### Synergy analysis

Mice were bled at day 1 and the serum expression of mAbs were analyzed by a human IgG ELISA. The serum was 2-fold serially diluted to generate a dose-response curve using cPASS (Genscript, NJ). The effective concentration (EC)_50_ was calculated for each individual mAb using CompuSyn (Paramux, NJ, USA). Serum from mice injected with a single HEDGES anti-SARS-CoV-2 mAb plasmid were combined in pairs at 2-times the EC_50_, and these combinations were run on cPASS. The combination index (CI) was calculated using CompuSyn where CI < 1, = 1 and >1 indicates synergism, additive effect and antagonism, respectively, as previously described [29–31] and as previously published by our group^43^. All CI values are calculated on the basis of the classic isobologram equation and assumptions [29–31].

### Statistical analysis

EC_50_ values were determined for each individual anti-SARS-CoV-2 mAb using CompuSyn (Paramux, NJ, USA). Comparisons were done in Excel using 2-tail t-tests, * p ≤ 0.05, ** p ≤ 0.01.

## Results

### Optimization of HEDGES DNA vectors following their intravenous administration into mice

We first assessed how large a DNA insert HEDGES DNA vectors can accommodate injection into immunocompetent mice. Recombinant-AAV (rAAV) vectors can only accommodate up to a 4.5 kb DNA insert [32]. In contrast, a HEDGES DNA vector containing a 7.2 kb DNA insert co-encoding the Mepolizumab heavy and light chain cDNAs plus the hG-CSF cDNA co-produced rapid then persistent therapeutic serum levels of all three genes following one intravenous injection [11]. Therefore, we next tested the potential effects on HEDGES DNA vector-based transgene cDNA encoded serum protein levels produced by intravenously administering either the 5J8 [13] cDNA (Fig 1a) or the hG-CSF [12] cDNA (Fig 1b). Each DNA vector contained DNA inserts ranging from 4 to 26.5 kb in size within an otherwise identical HEDGES DNA vector. Specifically, in the first experiment (Fig 1a), HEDGES DNA vectors encoding the 5J8 cDNA and containing DNA inserts ranging from 7.2 to 26.5 kb in size each produced comparable serum levels of the 5J8 protein (p > 0.05). In the second experiment, we intravenously injected HEDGES DNA vectors encoding the hG-CSF cDNA (Fig 1b) and containing DNA inserts ranging from 4 to 18 kb in size, also within otherwise identical HEDGES DNA vectors. Like 5J8, these hG-CSF cDNA encoding HEDGES DNA vectors containing various different DNA insert sizes also produced comparable serum levels of the hG-CSF protein (Fig 1b) (p > 0.05). In each experiment, the HEDGES plasmid DNA vector consisted of either one or multiple different expression cassettes. Each expression cassette contained the mouse CMV enhancer, EF1 promoter, SpaMaz [33], CTCF insulator [34] as well as the 5J8 or hG-CSF cDNA. Each expression cassette also contains varying numbers of BB which is a non-coding boundary sequence added to the 5’ or 3’ of the cassette. Therefore, unlike rAAV, DNA inserts of up to 26.5 kb do not limit HEDGES DNA vector transgene cDNA encoded serum protein levels produced following intravenous injection into immunocompetent mice. The capacity of HEDGES DNA vectors to accommodate much larger DNA inserts than rAAV further increases the number of human genes, including mAbs, that can be co-produced following intravenous HEDGES DNA vector injection.

### Creation of multiple expression cassette, single plasmid HEDGES DNA vectors

We next focused specifically on maximizing the ability of these novel HEDGES DNA vectors to co-produce multiple different transgene cDNA encoded human proteins in the serum of injected mice. Previously, one intravenous HEDGES DNA vector injection was shown to produce long-term therapeutic serum protein levels of either Mepolizumab or 5J8. Therapeutic serum ranges for mAbs are considered to be ≥ 1000 ng/ml. Therapeutic serum ranges for hGH and hGLA are > 1000 pg/ ml and for hG-CSF are > 100 pg/ ml [12–15,35,36] We then focused on creating a single HEDGES DNA vector plasmid with two expression cassettes. Each DNA expression cassette encoded unique mAb heavy and light chain cDNAs separated by a porcine self-cleaving peptide (P2A) DNA sequence [37]. We first tested one intravenous injection of a single HEDGES plasmid DNA vector co-encoding two different mAbs: Mepolizumab and 5J8 (Fig 1c). Intravenous injection of this HEDGES DNA vector co-produced therapeutic serum levels of Mepolizumab as well as 5J8. These results demonstrate that one intravenous HEDGES DNA vector injection co-produces persistent therapeutic serum levels of two different mAbs.

We then tested how many different injected genes could co-produce therapeutic serum levels of their transgene cDNA encoded human proteins following one intravenous HEDGES DNA vector injection into immunocompetent mice. Specifically, we tested single HEDGES DNA vectors containing four, six, or eight different heavy and light chain cDNAs of mAbs within two, three, or four separate expression cassettes, respectively. Mice injected with the HEDGES DNA vector encoding four different mAbs (the anti-human CD20 mAb Rituximab [14], Mepoluzimab [15], 5J8 [13], and the anti-SARS-COV2 mAb H4 [38]) co-produced therapeutic serum protein levels of three of these four different mAbs (all except Rituximab) (Fig 1d).

To attempt to further increase both the level and the duration of transgene cDNA encoded serum mAb proteins over time, we then tested the potential effects of the specific enhancer element incorporated. Specifically, we compared the relative effects of the murine CMV (mCMV) versus the human CMV (hCMV) enhancer [39]. We also directly compared the effects of placing the enhancer 5’ versus 3’ to each of the two expression cassettes within the HEDGES DNA vector. We found no statistically significant difference was produced by placement of the human versus murine CMV enhancer [39] in driving cDNA encoded serum protein production levels (Fig 1e). While expression of both mAbs driven by mCMV appears higher those driven by hCMV, regardless of whether they are positioned in the first or second expression cassette, only 5J8 driven by mCMV in the first expression cassette produced statistically significantly higher serum levels than hCMV (Fig 1e).

### Co-injection versus repeat-injection of HEDGES DNA vectors

We then compared two different strategies for expressing at least seven different cDNA encoded serum protein products. Our first approach was a single co-injection of four different plasmids, each encoding two different mAb or non-mAb proteins. We detected simultaneous co-production of seven different transgene cDNA encoded proteins, including Rituximab, Mepoluzimab, 5J8, anti-SARS CoV2, hGLA, hGH, and hG-CSF twenty-four hours after injection (Fig 2a). The serum levels produced were largely within their respective therapeutic ranges [12–15,27,28]. Our second approach was to perform four injections over a four-week period. Each week a single DNA vector co-encoding two different genes was injected. By day 22 after four injections, a total of eight transgene cDNA encoded proteins, Rituximab, Mepolizumab, 5J8, B38 (anti-SARS-CoV-2), hGLA, hG-CSF, hGH, and ACE-2 were detected (Fig 2b). It demonstrated that multiple different proteins (up to 8 different proteins) could be co-produced by either simultaneous or sequential adminstration via the HEDGES platform.

### Measurement of potential synergy produced by combinations of two different anti-SARS-CoV-2 mAbs

**Fig 3:**
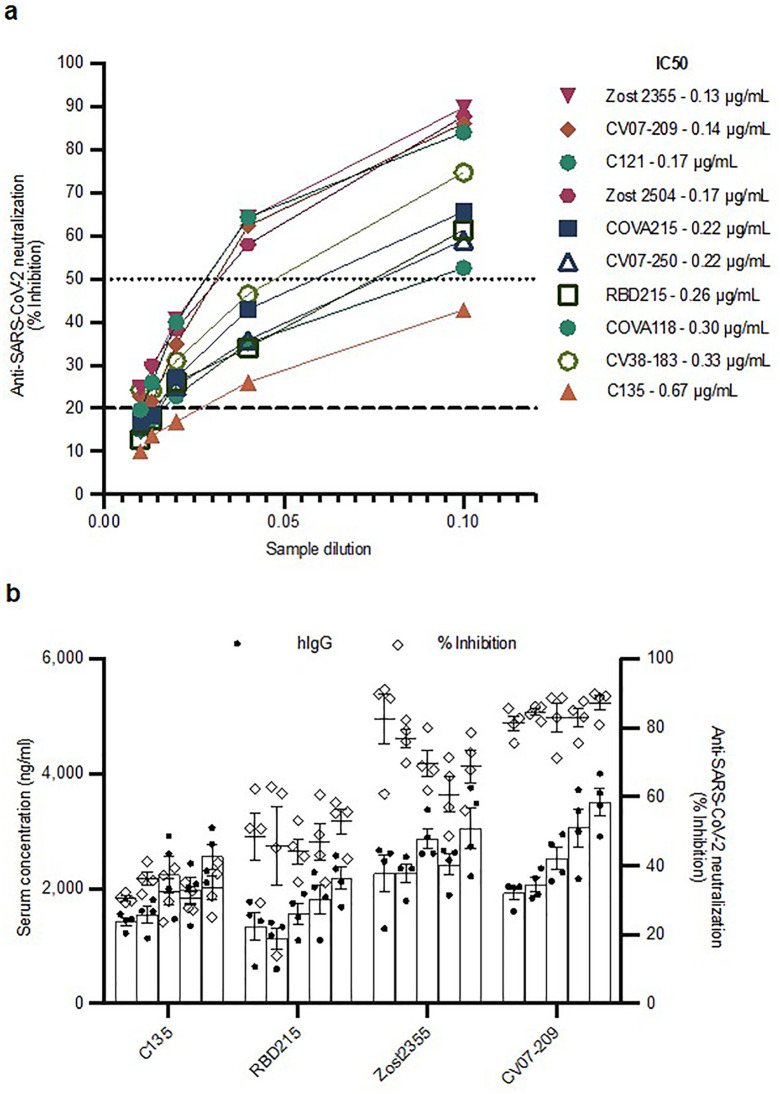
HEDGES expression of anti-SARS-CoV-2 mAbs. **a** Surrogate neutralization levels of each anti-SARS-CoV-2 mAb 24 hours after IV HEDGES injection of mice in groups of 3. Samples were run at 1:10, 1:25, 1:50, 1:75, and 1:100. Values are the mean of two cPass technical replicates. The IC50 as shown was calculated via GraphPad Prism 9. **b** hIgG ELISA (bars, solid circles: left y-axis) and surrogate neutralization (open diamonds: right y-axis) data for mice after receiving IV HEDGES with one of four different anti-SARS-CoV-2 mAbs. Values are mean ± SEM, n = 4, each bar represents days 1, 8, 22, 30, and 36 from left to right.

Drug combinations are a powerful tool often used to treat cancer and infectious diseases among others [29–31]. Identifying synergistic anti-SARS-CoV-2 mAb combinations is desirable and should maximize the power of this HEDGES platform. Before determing the anti-SARS-CoV-2 mAb combination we administered, we measured the SARS-CoV2 neutralization ability of each individual anti-SARS-CoV-2 mAb using cPASS. The inhibition mAb produced (Fig 3a) is in accordance published data [24–28]. To confirm their anti-SARS-CoV-2 mAb co-production in mice, four different HEDGES DNA encoded were IV administered in mice. Therapeutic serum levels of each anti-SARS-CoV-2 mAb were rapidly then durably produced with corresponding neutralizing activity (Fig 3b).

### Rapid and durable co-production of up to ten different anti-SARS-CoV-2 mAbs following intravenous HEDGES DNA vector injections

**Fig 4:**
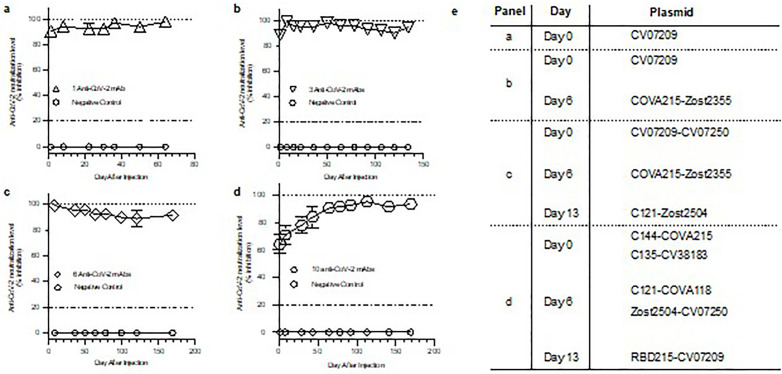
Co-production of up to ten different anti-SARS-CoV-2 mAbs following intravenous HEDGES DNA vector injections. a-d.

We next assessed how many different anti-SARS-CoV-2 mAbs could be co-produced in immunocompetent mice. Specifically, we measured the total anti-SARS-CoV-2 surrogate serum neutralization levels co-produced over time following HEDGES DNA vector-based administration of a total of one, three, six, or ten different anti-SARS-CoV-2 mAbs in immunocompetent mice (Fig 4a–4d). We first tested intravenous injection of one HEDGES DNA vector encoding a single anti-SARS-CoV-2 mAb cDNA (CV07209 [25] in groups of four mice. The injection schedule with specific plasmids is outlined in Fig 4e for all panels. One anti-SARS-CoV-2 mAb cDNA injection produced 90% SARS-CoV-2 mAb virus surrogate neutralization activity at day one, 98% surrogate neutralization at day 64(Fig 4a).

We then tested the surrogate serum neutralization of three different anti-SARS-CoV-2 mAbs. We intravenously injected a group of three mice one HEDGES DNA vector encoding one anti-SARS-CoV-2 mAb cDNA CV07−209 [25]) on day zero, followed by IV injection of one HEDGES DNA vector co-encoding two different anti-SARS-CoV-2 mAbs (COVA215 [24] and Zost2355 [28]) on day seven (Fig 4e). Thus, a total of three different anti-SARS-CoV-2 mAbs were expected to be co-produced. These three mAbs coproduced 89% SARS-CoV-2 mAb surrogate serum neutralization at day one and 95% surrogate neutralization at day 134 (Fig 4b).

Next, we tested six different anti-SARS-CoV-2 mAbs surrogate serum neutralization following an IV HEDGES injection schedule of one administration per week for three weeks in groups of four mice. First, we intravenously co-injected of two HEDGES DNA vectors co-encoding a total of four different anti-SARS-CoV-2 mAb cDNAs CV07−209 [25]) on day zero, followed by IV injection of one HEDGES DNA vector co-encoding two different anti-SARS-CoV-2 mAbs (COVA215 [24] and Zost2355 [28]) (CV07−209 [25] and CV07−250 [25] plus COVA215 [24] and Zost2355 [28]), then followed seven days later by IV injection of one HEDGES DNA vector co-encoding two different anti-SARS-CoV-2 mAb cDNAs (C121 [27] and Zost2504 [28]) (Fig 4e). At three weeks, six different anti-SARS-CoV-2 mAb cDNAs (Fig 4c) co-produced 96% SARS-CoV-2 virus surrogate neutralization and 92% surrogate neutralization at day 169.

Finally, to attempt to co-produce a total of ten different anti-SARS-CoV-2 mAbs, we followed an IV HEDGES injection schedule of one injection per week for three weeks. Specifically, the first week co-injected two different dual expression cassette HEDGES DNA vectors encoding a total of four different anti-SARS-CV07–209 [25]) on day zero, followed by IV injection of one HEDGES DNA vector co-encoding two different anti-SARS-CoV-2 mAbs (COVA215 [24] and Zost2355 [28])CoV-2 mAbs (C144 [27] and COVA215 [24], CV38−183 [25] and C135 [25] on day 0) and the on second week, we co-injected two additional dual expression cassette HEDGES DNA vectors (C121 [27] and COVA118 [24], and Zost2504 [28] and CV07−250 [25] on day 6). These co-injections were followed by a third injection on day 13 of one dual expression cassette HEDGES DNA vector encoding two different anti-SARS-CoV-2 mAbs (RBD215 [26] and CV07−209 [25]). Thus together, we injected a total of five different HEDGES DNA vectors encoding a total of ten different anti-SARS-CoV-2 mAb cDNAs via three once a week injection (Fig 4e). These co-injections coproduced 64% SARS-CoV-2 mAb virus surrogate neutralization at day one and 94% surrogate serum neutralization activity at day 169 (Fig 4d). Taken together, these results document that intravenous, HEDGES DNA vector-based injection of one to ten different anti-SARS CoV-2 mAbs routinely produced ≥ 90% SARS-CoV-2 surrogate serum neutralization activity.

### HEDGES co-produces rapid onset, sustained co-production of twelve different anti-pandemic mAbs

Fig 4 shows the results of co-administering HEDGES DNA vectors co-encoding up to ten different anti-SARS-CoV-2 mAbs. To measure total SARS-CoV-2 surrogate serum neutralizing levels co-produced, individual mouse sera were simultaneously quantitated for their ability to inhibit RBD binding (the cPASS assay [16]), as well as by measuring total human IgG (h-IgG) serum levels. However, using the cPASS assay together with the human IgG assay does not enable measurement of serum levels of the ten different individual anti-SARS-CoV-2 mAbs. This is because neither RBD binding inhibition nor hIgG serum levels can specifically differentiate individual serum levels of any of these different anti-SARS-CoV-2 mAbs [16–19]. Thus, the precise serum levels of any of the ten different individual anti-SARS-CoV-2 mAbs could not be assayed individually by the assays used in Fig 4.

**Fig 5:**
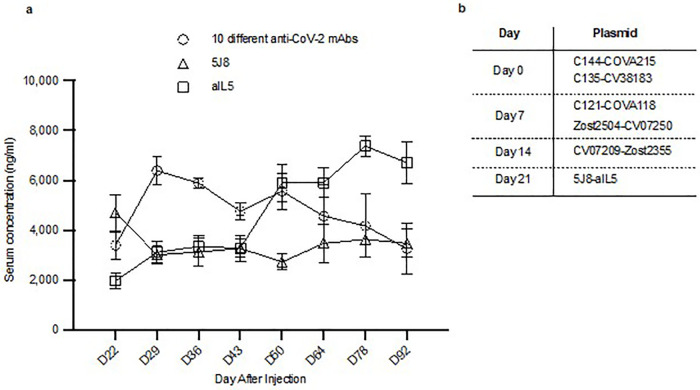
Demonstrating that HEDGES co-produces twelve different mAbs. **a** ELISA values from aCoV-2 (open circles), aIL5 (open squares), and 5J8 (open triangles) specific ELISAs starting at day 22 up to day 92. Values are mean ± SEM, n = 5. **b** Injection schedule for panel a. Mice were IV-injected with two HEDGES plasmid DNA vectors encoding a total of four different anti-SARS-CoV-2 mAbs on week 1, two HEDGES plasmid DNA vectors encoding a total of four different anti-SARS-CoV-2 mAbs on week 2, one HEDGES plasmid DNA vector encoding a total two different anti-SARS-CoV-2 mAbs on week 3, and one HEDGES plasmid DNA vector encoding both Mepolizumab (aIL5) and 5J8 on week 4.

Therefore, we then repeated the three weekly intravenous HEDGES DNA vector injections that co-produced ten different anti-SARS-CoV-2 mAbs, exactly as performed in Fig 4D above in Fig 5A. However, in Fig 5A, these three weekly intravenous injections were followed by a fourth intravenous HEDGES DNA-vector injection. The fourth HEDGES DNA vector injected co-encoded 5J8 and Mepolizumab. We specifically co-injected a HEDGES DNA vector co-encoding these two mAbs because serum levels of the 5J8 and Mepolizumab mAbs can each be individually measured using individual, mAb specific ELISA assays [13,15]. Concurrently, we measured the total SARS-CoV-2 neutralizing serum levels co-produced by all ten different anti-SARS-CoV-2 mAbs using the cPASS assay.

By measuring 5J8 as well as Mepolizumab serum levels using their mAb specific ELISAs, we found that both the 5J8 and Mepolizumab serum levels co-produced by the fourth co-injection were directly comparable to the 5J8 as well as Mepolizumab serum levels previously produced by a single intravenous injection of one HEDGES DNA-vector encoding either 5J8 [13] or Mepolizumab [15]. Taken together, the results shown in Fig 5 document our hypothesis that the HEDGES platform retains full mAb co-production efficacy following a fourth HEDGES DNA-vector injection co-encoding two different mAbs, each of which can be quantitated by a specific ELISA.

### Production of highly neutralizing anti-SARS-CoV-2 mAb based serum activity within twenty hours following one HEDGES DNA vector injection

**Fig 6:**
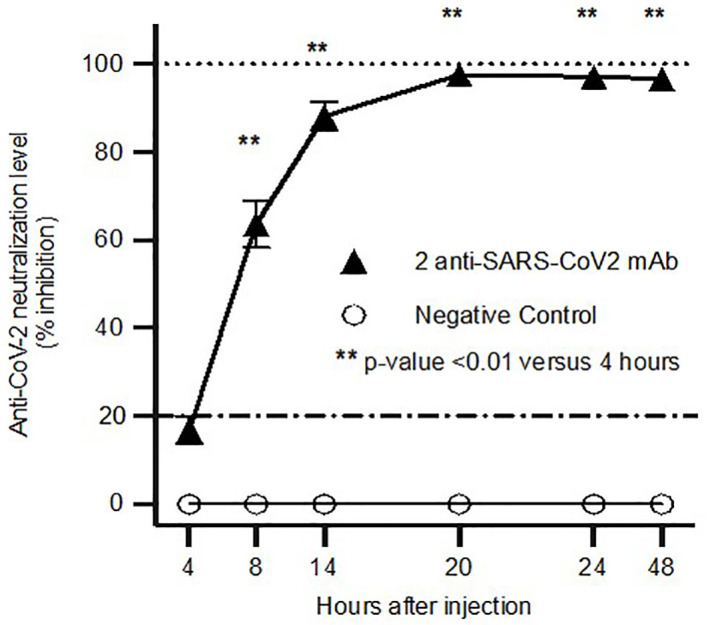
Time course of anti-SARS-CoV-2 surrogate serum neutralizing following one HEDGES DNA vector injection. Surrogate serum neutralization data from various time points (hours) after mice (mean ± SEM, n = 4) were injected one IV HEDGES dual expression plasmid. 5 groups of 4 mice were injected with each plasmid. One group of mice was bled at each timepoint shown. The same group of mice were bled for the 4- and 18-hour timepoints, the 8- and 20-hour timepoints, and the 24- and 48-hour timepoints. In each case, the second bleed was a terminal bleed. Statistical significance is indicated with an asterisk as p ≤ 0.01 against 4 hours post injection.

It can take from weeks to months to produce maximal anti-SARS-CoV-2 infection protection following anti-SARS-CoV-2 vaccine administration^9^. Therefore, we measured the anti-SARS-CoV-2 surrogate serum neutralization activities co-produced by one intravenous HEDGES DNA vector injection co-encoding two different anti-SARS-CoV-2 mAbs on day zero, followed by IV injection of one HEDGES DNA vector co-encoding two different anti-SARS-CoV-2 mAbs (COVA215 [24] and Zost2355 [28]) (CV07−209 [25] and COVA215 [24]) between 4- and 48-hours post injection. Specifically, individual groups of four mice were bled at either 4, 8, 14, 20, 24, or 48 hours after one HEDGES DNA vector injection. The first blood draw was performed for each group at 4, 8, or 14 hours and the second blood draw happened at euthanasia for 20, 24, or 48 hours. The data from all of the groups of mice was used together to generate a single graph representing the rise of surrogate neutralization that occurs within 48 hours post HEDGES IV injection. When compared to sera from control uninjected mice which showed background control serum levels, mice bled at the above time points showed 17, 64, 88, 98, 97, and 97 percent inhibition of RBD binding, respectively (Fig 6). The surrogate neutralizing activities of mouse sera harvested at 8 hours or later after injection were all statistically significantly different (p < 0.01) when compared to sera harvested 4 hours after HEDGES DNA vector injection. Therefore, intravenous injection of one HEDGES DNA vector co-encoding two different anti-SARS-CoV-2 mAbs produced 98% SARS-CoV-2 surrogate neutralization activity within 20 hours post injection.

For large scale rodent studies, to create, upscale, deploy, then administer new HEDGES DNA vectors encoding new anti-pandemic mAbs requires approximately two weeks.

For large scale human administration, to create, upscale, deploy, then administer new bioreactor produced recombinant anti-pandemic mAbs requires approximately 12 weeks.

Drug combinations are a powerful tool often used to treat cancer and infectious diseases among others [29–31]. Identifying synergistic anti-SARS-CoV-2 mAb combinations is desirable and should maximize the power of this HEDGES platform. We tested every iteration of two mAb combinations from the ten different anti-SARS-CoV-2 mAbs by analyzing the serum from mice that were injected with a single HEDGES DNA vector encoding one of the ten different anti-SARS-CoV-2 mAbs. This approach allowed us to determine which anti-SARS-CoV-2 mAb combinations produced synergistic, additive, or antagonistic effects on the overall level of anti-SARS-CoV-2 surrogate serum neutralization activity produced. We found that most of these two different anti-SARS-CoV-2 mAb combinations produced synergistic levels of anti-SARS-CoV-2 virus surrogate serum neutralization (Table 1). A total of 45 different combinations of two different anti-SARS-CoV-2 mAb combinations were analyzed. We identified CV07−250 [25] and C144 [27] as the most synergistic pair with a CI_0.75_ value of 0.57, while Zost2355 [28] and Zost2504 [28] with a CI_0.75_ value of 1.48 was the most antagonistic pair. A CI_0.75_ value of < 1, = 1 and > 1 indicates synergism, additive effect, and antagonism, respectively. A representative sample of this data is shown in Table 1. Overall, this HEDGES based anti-SARS- CoV-2 mAb approach synergistically enhanced inhibition of RBD-ACE2 binding activity in the majority of different two anti-SARS-CoV-2 two mAb combinations tested.

**Table 1:** Synergistic neutralization activity demonstrated by the majority of combinations of two different anti-SARS-CoV2 mAbs. The combination index (CI) was calculated at a concentration predicted to produce 75% surrogate neutralization using Compusyn, where CI < 1, = 1 and > 1 indicates synergism, additive effect, and antagonism, respectively, as previously described based on the classical isobologram equation. The numbers 1-12 indicate one antibody pair from a total of 45 pairs tested.

Combinations of 2 mAb	CI0.75	Relationship
1	0.57	Synergistic
2	0.58	
3	0.70	
4	0.71	
5	0.83	
6	0.85	
7	0.88	
8	0.89	
9	0.93	Additive
10	1.00	
11	1.08	
12	1.48	Antagonistic

## Discussion

We have previouslydemonstrated in a prior publication introducing HEDGES that it has strong efficacy as well as safety profiles in mice [11]. Specifically, from an efficacy perspective, we demonstrated that one IV HEDGES DNA-vector administration into immunocompetent mice safely, rapidly then durably produces therapeutic serum levels of one or more cDNA encoded human proteins. These include rapidly then durably producing therapeutic serum levels of the FDA-approved human cytokine, hG-CSF (protein T1/_2_ ~ 2 hours [11,12]. HEDGES also produces durable therapeutic serum levels of 5J8, an anti-1918 pandemic influenza mAb [13], Rituximab, an anti-human CD20 mAb [14], and Mepolizumab, an anti-human IL-5 mAb [15]. HEDGES cDNA vector-encoded proteins are produced in ~35% of all lung vascular endothelial cells [11]^,^. From a safety perspective, we showed that HEDGES neither detectably integrates into genomic DNA, induces adaptive immune responses, nor elicits anti-vector targeted immune responses that prevent effective re-dosing in immunocompetent hosts [11]. In addition, critical rodent toxicity markers remain near or at background levels [11].

Herein we show that 4 weekly intravenous HEDGES DNA vector administrations into immunocompetent mice effectively, rapidly, and durably co-produce twelve different, highly neutralizing anti-pandemic mAbs. These include ten different anti-SARS-CoV-2 mAbs [24–28] plus 5J8, an anti-1918 pandemic influenza mAb [13] and the anti-human IL-5 immunomodulatory mAb, mepolizumab [15]. HEDGES co-produces highly neutralizing serum levels of 12-different anti-pandemic mAbs for > 93 mouse days in immunocompetent mice. This is the equivalent of greater than an estimated 10 years in humans based on an average mouse-human lifespan conversion [40]. (The mouse-human equivalency is an estimate that fluctuates across multiple variables including lifespan and mouse strain and is used strictly as an estimate to compare the data from mice to humans). Since each intact mAb requires individual heavy and light chain cDNAs, this same HEDGES generation-2 platform can produce a total of 24 individual cDNA-encoded proteins (Table 2). Alternatively, it can co-produce eight different anti-pandemic mAbs together with eight different cDNA co-encoded individual proteins. For example, this would allow combining multiple different, each highly synergistic, highly-neutralizing anti-pandemic mAbs together with recombinant, soluble ACE-2 (rACE-2) protein. rACE-2 protein has been shown to exert significant anti-SARS-CoV-2 neutralizing activity, as well as anti-ARDS activity [38]. In addition, these HEDGES generation-2 platform results document that each anti-pandemic mAb included in these HEDGES-based combinations can be precisely selected to render the anti-pandemic mAb combinations co-produced maximally synergistic (Table 1). As such, HEDGES may offer multiple advantages over anti-pandemic vaccines as well as bioreactor-produced mAbs [10].

**Table 2:** Critical anti-pandemic disease platform characteristics HEDGES versus recombinant mAbs versus vaccines.

Critical platform characteristics	HEDGES	Recombinant mAbs	Vaccines
Number of different, highly selected, highly neutralizing anti-pandemic mAbs produced	≥12 (Fig 6)	≤2 [4]	Not applicable (NA)
Produces highly selected, highly synergistic combinations of mAbs	Yes (Table 1)	No [4]	NA
Onset of fully neutralizing activity	<24 hours (Fig 5)	<24 hours [4]	Weeks to months [9]
Duration of fully neutralizing activity produced following single administration	Durable (Fig 6)	~3 weeks [4]	Weeks to months [9]
Requires an intact cold chain	No [10]	Yes [4]	Yes [9]
Can be freeze dried	Yes [41]	No [4]	No [9]
Time from creation to deployment	~3 weeks (Fig 7)	~9 months [10]	~3 months [9]

Anti-pandemic vaccines cannot selectively produce highly-synergistic, highly-neutralizing combinations of anti-pandemic mAbs [9,42]. Furthermore, anti-pandemic bioreactor-manufactured mAbs can produce highly neutralizing serum levels of a maximum of only two different anti-SARS-CoV-2 mAbs per person [43]. To date, combinations of a maximum of two different highly selected anti-SARS-CoV-2 mAbs are unable to prevent the development of pan-resistant SARS-CoV-2 escape mutant strains [44]. Conversely, HEDGES now co-produces combinations of at least twelve different anti-pandemic mAbs in immunocompetent mice (Fig 5). These mAbs include ten different anti-SARS-CoV-2 mAbs [24–28] 5J8, plus an anti-pandemic influenza mAb [13] and mepolizumab, an immunomodulatory anti-human IL-5 mAb [15]. Thus, HEDGES now co-produces twelve different anti-pandemic mAbs that can effectively, rapidly and durably co-protect against SARS-CoV-2 and pandemic influenza infections (Fig 5).

Previously, the role of anti-viral drug synergy in transforming incurable viral diseases into either a chronic or curable disease has already been clearly demonstrated. This is documented by highly selected combinations of at least three different anti-retroviral drugs transforming HIV from a fatal into a chronic disease, as well as transforming hepatitis C from an incurable into a largely curable disease [20–22,45,46]. Importantly, the majority of combinations of two different HEDGES-produced anti-SARS CoV-2 mAbs we tested produced synergistic anti-SARS-CoV-2 serum neutralizing levels (Table 1). These HEDGES based synergy results suggest that using sophisticated anti-SARS-CoV-2 mAb receptor binding domain (RBD) binding site mapping studies will enable the creation of at least ten different HEDGES anti-SARS-CoV-2 mAbs, each precisely programmed to bind the SARS- CoV-2 virus RBD at a unique, non-overlapping binding site [28,47]. This approach should enable each of these ten different HEDGES anti-SARS-CoV-2 mAbs to bind the RBD in a non-overlapping manner. Such a precisely programmed, highly combinatorial approach may enable non-overlapping anti-SARS-CoV-2 mAb saturation binding of the RBD, potentially preventing SARS-CoV-2 virus infection against otherwise now pan-resistant SARS-CoV-2 escape mutant virus strains [48].

The ongoing evolution of progressively more pan-resistant SARS-CoV-2 virus escape mutant virus strains is clearly illustrated by the progressive development of SARS-CoV-2 Omicron strains now pan-resistance to all available anti-SARS-CoV-2 vaccines as well as to anti-SARS- CoV-2 mAbs [3,6,42,49,50]. This progressive pan-resistance is documented by the ongoing inactivation of anti-SARS-CoV-2 mAbs that previously effectively prevented as well as treated pre-Omicron SARS-CoV-2 virus strains in humans when administered either as single anti-SARS-CoV-2 virus mAbs or in combinations of two different anti-SARS-CoV-2 mAbs. Subsequently, and after the FDA-approved the anti-SARS-CoV-2 mAb combinations bamlanivimab and etesevimab [4], as well as casirivimab plus imdevimab [51,52] became ineffective against the later Omicron strains, sotrovimab remained effective against them. However, subsequently emerging Omicron virus escape mutant virus strains eventually became resistant to sotrovimab [53]. Based on this ongoing pattern of even more pan-resistant Omicron virus escape mutant strains continuing to emerge, the ability to co-administer many more than two-different anti-SARS-CoV-2 mAbs may prove essential essential for preventing the ongoing emergence of evermore pan-resistant escape mutant-strains.

HEDGES’s abilities to rapidly then durably co-produce highly neutralizing serum levels of 10-different anti-SARS-CoV-2 mAbs) as well as highly neutralizing serum levels of 5J8, an anti-1918 pandemic influenza mAb [13], a total of 11-different anti-pandemic mAbs, together with mepolizumab [15], an immunomodulatory mAb (Fig 5) may effectively prevent the subsequent emergence of otherwise now pan-resistant SARS-CoV-2 virus escape mutant strains, as well as potentially prevent the emergence of other novel pandemic diseases that may subsequently emerge at any time. HEDGES can also incorporate novel anti-SARS-CoV-2 mAbs, including functionally Fc- modified mAbs [54,55] as well as bi-specific mAbs [56,57]. into combinations of at least ten different anti-SARS-CoV-2 mAbs. Functional Fc receptor domain modification has been shown to improve treatment outcomes in SARS-CoV-2-infected mice [58]. These results indicate that the presence of functionally Fc modified mAbs incorporated into selected, HEDGES based SARS- CoV-2 mAb combination regimens may effectively treat already severely ill, SARS-CoV-2 infected human patients. To date, these patients have remained largely treatment refractory [58]. These results also suggest that incorporating selected, functionally Fc-modified anti-SARS-CoV-2 mAbs and/or bi-specific mAbs may also act synergistically when included in combinations of ten different anti-SARS-CoV-2 mAbs.

**Fig 7:**
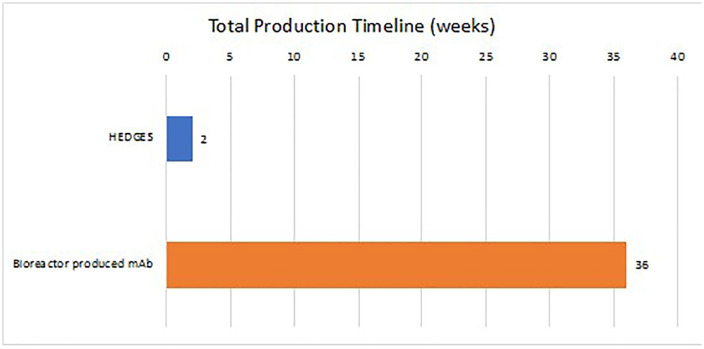
Respective timelines for production of new HEDGES DNA-vector encoding anti-pandemic mAbs for large scale rodent administration versus production of new bioreactor produced recombinant anti-pandemic mAbs for large scale human administration. Production time in days from the start of development. The HEDGES timeline includes the time necessary to optimize, create, sequence verify, and produce each plasmid. The recombinant mAb timeline includes the time necessary to develop and produce each mAb.

Furthermore, vaccines as well as bioreactor-produced mAbs require an intact cold chain, thus constraining their deployment and increasing costs [43,59]. Conversely, HEDGES can create, upscale then widely deploy one or more new, more effective anti-pandemic mAbs in <3 weeks after their identification (Fig 7). This is at least in part because neither HEDGES DNA vectors nor liposomes, HEDGES only two components, require an intact cold chain [59] (Further testing under GMP conditions is crucial). Of critical importance, HEDGES can readily be freeze dried. This enables its prolonged storage at ambient temperatures, even in equatorial regions worldwide [41].

We exclusively used immunocompetent CD-1 mice in these studies, together with HEDGES DNA vectors that encode partially or fully humanized proteins. As shown previously, following one IV injection of a HEDGES DNA vector encoding rituximab [14], a largely humanized FDA-approved mAb elicits highly neutralizing, mouse anti-human protein antibody responses in from one quarter to one third of CD-1-injected mice over time [11]. The onset of a highly neutralizing mouse anti-human antibody response produces an interspecies artifact that then rapidly reduces HEDGES cDNA encoded mAb serum protein levels towards background levels [11]. Therefore, HEDGES should prove even more effective in humans than in immunocompetent mice.

To determine whether HEDGES strong, consistent efficacy as well as safety profiles it demonstrates in mice are reproduced in humans will first require completing a rigorous, FDA-supervised, Investigational New Drug Application (INDA) large animal-based toxicity studies. These studies will include detailed, short and long-term histopathologic as well as extensive blood analyses to assess whether HEDGES causes significant either short and/or long-term toxicity in large animals that best predict the subsequent occurrence of toxic effects in humans. Since HEDGES involves intravenous administration of a DNA vector, these studies will also include the sensitive determination of whether the DNA-vector integrates into host genomic DNA, with special attention to host germline tissues.

If HEDGES successfully completes these INDA studies without causing significant either short or long term large animal toxicity, together with the absence of detectable DNA vector integration into host germline tissues, this will enable a HEDGES-based phase 1 human clinical trial. A critical component of a HEDGES phase 1 human clinical trial is to determine its MTD or Maximal Tolerated Dose. The HEDGES MTD is the highest HEDGES dose that can be given without causing unacceptable toxicity in the person. Overall, a HEDGES phase 1 clinical trial would be conducted to determine whether HEDGES is also safe in humans, as well as collecting preliminary data as to whether it is able to prevent SARS-CoV-2 infection.

Taken together, it is possible our new, highly combinatorial, highly synergistic, HEDGES-generation-2 anti-pandemic mAb-based platform may effectively, rapidly then durably co-prevent now otherwise pan-resistant SARS-CoV-2 escape mutant virus strains^6^. By designing synergistic combinations of many different anti-SARS-CoV-2 mAbs, it may even be possible to prevent SARS-CoV-2 infection, even in severely immunosuppressed individuals. In addition, this HEDGES-generation-2 anti-pandemic platform may effectively then durably co-prevent pandemic influenza [13], HIV [20–22], and/or malaria [23], as well as new, even more-transmissible, -pan resistant and/or -lethal pandemic diseases that may subsequently emerge at any time [1,2]. Overall, HEDGES may enable progressively more precisely targeted modification of its HEDGES human DNA vector-based platform to prevent as well as treat an expanding array of now difficult or impossible to prevent or treat human diseases.
